# Research on the Regulatory Effects of Resveratrol on BCG-Induced Autophagy and Apoptosis in Macrophages via Targeting SIRT1

**DOI:** 10.3390/microorganisms14102365

**Published:** 2026-10-15

**Authors:** Ruiqian Li, Yujie Xie, Mingzhu Li, Wenhao Wan, Chang Xu, Yu Sun, Xiaoliang Wang, Guangcun Deng, Lihua Xu

**Affiliations:** 1 College of Animal Science and Technology, Ningxia University, Yinchuan 750021, China; 2 College of Life Science, Ningxia University, Yinchuan 750021, China; 3 Key Lab of Ministry of Education for Protection and Utilization of Special Biological Resources in Western, Ningxia University, Yinchuan 750021, China; 4 Ningxia Hui Autonomous Region Animal Disease Prevention and Control Center, Yinchuan 750011, China

**Keywords:** resveratrol, *Bacillus Calmette-Guérin*, macrophage, SIRT1 pathway, autophagy, apoptosis

## Abstract

*Mycobacterium tuberculosis* (*Mtb*) infection can trigger immune responses in macrophages during its autophagy and apoptosis, as two crucial cellular processes which are essential for the host to clear the pathogen. Resveratrol (RES) is a natural polyphenolic compound with various biological functions such as anti-inflammatory, antioxidant, and immunomodulatory activities. However, the specific mechanism by which it regulates autophagy and apoptosis in a *Bacillus Calmette-Guérin* (BCG)-infected macrophage model remains to be elucidated. This study aimed to analyze the effects of RSE on BCG-induced autophagy and apoptosis in macrophages and explore the underlying molecular mechanisms. The mouse macrophage cell line RAW264.7 was used to establish a BCG infection model and evaluate the interventional effects of RES. Cell viability was detected by CCK-8 assay. The expression levels of autophagy- and apoptosis-related proteins were analyzed by Western blotting. LC3 puncta formation was observed by immunofluorescence. Autophagy rate and apoptosis rate were determined by flow cytometry. The ultrastructure of autophagosomes and autolysosomes was observed by transmission electron microscopy. The results showed that BCG infection significantly increased the expression of LC3-II, Beclin1, Atg5, and Atg7 in macrophages; decreased p62 levels; and increased the number of intracellular autophagosomes and autolysosomes. Concurrently, BCG infection also induced apoptosis, including upregulation of Bax, Caspase 3, Caspase 9, and PARP1 expression; downregulation of Bcl-2 expression; and an increased apoptosis rate. RES promoted autophagosome accumulation and inhibited apoptosis in BCG-infected macrophages in a concentration-dependent manner; however, these effects were significantly attenuated after SIRT1 gene silencing by siRNA and inhibition by EX527. Therefore, it is speculated that RES may regulate autophagy and apoptosis through the SIRT1/FOXO1 pathway. These findings provide a potential experimental basis for developing RES into a host-directed therapeutic (HDT) agent, and offer new insights into the mechanisms by which natural products participate in host defense.

## 1. Introduction

Tuberculosis is a chronic, consumptive infectious disease affecting both humans and animals caused by *Mycobacterium tuberculosis* (*Mtb*) [1]. According to the World Health Organization’s Global Tuberculosis Report 2024, there were 10.8 million new tuberculosis cases globally in 2023, including 400,000 cases of multidrug-resistant tuberculosis (MDR-TB) [2]. On World Tuberculosis Day in 2022, WHO highlighted that the COVID-19 pandemic has reversed years of global progress in the fight against tuberculosis. Currently, issues such as drug resistance and multidrug resistance pose significant challenges to achieving control over the disease’s development [3,4].

*Mtb* is an intracellular pathogen. As the primary host cells, macrophages can initiate various defense mechanisms, including autophagy and apoptosis, to combat *Mtb* infection [5]. Recent studies have revealed complex crosstalk between autophagy and apoptosis, which collectively regulate the outcome of *Mtb* infection. *Mtb* has evolved multiple strategies to counteract host defenses by manipulating effector mechanisms such as phagosome maturation, vacuolar escape, autophagy, antigen presentation, and metabolic pathways, enabling pathogenic *Mtb* to establish long-term infection and persist within host cells [6]. The first-line anti-tuberculosis drugs currently used clinically, such as rifampicin and isoniazid, were developed in the last century. While effective, they suffer from drawbacks including long treatment duration, poor patient compliance, significant adverse effects, and unsatisfactory results in trials combining multiple drugs to shorten treatment time [7,8]. Consequently, there is an urgent need for therapeutic regimens with shorter durations, higher efficacy, better safety, and improved tolerability [9,10,11]. In this context, a host-directed therapy (HDT) approach, which targets the host response, has emerged as an adjunctive treatment for tuberculosis [12,13]. Compared to conventional chemotherapy, HDT targets the host’s complex defenses, aiming to enhance protective immune responses, suppress excessive inflammation, improve the host’s ability to maintain homeostasis, and increase the efficacy of chemotherapy [14,15,16,17]. Since HDT does not directly target *Mtb* itself, it is less likely to induce drug resistance [14,16]. Resveratrol (RES), a natural polyphenolic compound and SIRT1 agonist, is a bioactive ingredient found in plants such as Polygonum cuspidatum, grapes, and peanuts. It possesses various biological functions, including anti-inflammatory, antioxidant, immunomodulatory, antiviral, and antibacterial activities [18,19,20]. In recent years, RES has garnered significant attention in biomedical research due to its favorable pharmacological properties and notable ameliorative effects on various diseases [21]. However, whether and how RES regulates the processes of autophagy and apoptosis in Bacillus Calmette-Guérin (BCG)-infected macrophages remains incompletely understood. This study aims to systematically investigate the regulatory effects of RES on BCG-induced autophagy and apoptosis in macrophages and to elucidate the underlying molecular mechanisms. The results of this study only explore the pharmacological effects of RES using RAW264.7 cells infected with the vaccine strain BCG, and have not been applied to virulent *Mtb* strains, thus providing a potential theoretical basis for research on HDT for tuberculosis.

## 2. Materials and Methods

### 2.1. Cell Culture

RAW264.7 cells were purchased from the Shanghai Cell Bank of the Chinese Academy of Sciences (Shanghai, China). The cells were cultured in DMEM complete medium (Gibco, Grand Island, NY, USA) supplemented with 10% fetal bovine serum (Thermo Scientific, Waltham, MA, USA). When the cell density reached 80–90%, the culture medium was discarded and the cells were washed three times with PBS. Subsequently, the cells were digested with 0.25% trypsin for 2 min, after which the digestion was terminated by adding complete medium. The cells were then centrifuged at 900 r/min for 5 min to collect the cell pellet. After resuspension, the cells were seeded into 10 cm culture dishes at a density of 8 × 10^6^ cells per dish and incubated at 37 °C in a humidified incubator with 5% CO_2_ for 24 h.

### 2.2. Bacterial Culture

The BCG strain is an attenuated variant derived from a virulent strain of *Mycobacterium bovis* through serial passaging. The BCG strain used in this study (Batch No.: D2-JIA12-1) was purchased from the Shanghai Institute of Biological Products (Shanghai, China). BCG was cultured in Middlebrook 7H9 liquid medium supplemented with 600 μL Tween-80. Following autoclaving, inoculation, and single-colony isolation, the bacteria were cultured at 37 °C with 5% CO_2_ until the OD600 nm reached 1.5. Bacterial cells were collected by centrifugation, resuspended in complete medium, and adjusted to the desired concentration. RAW264.7 cells were seeded at a density of 1 × 10^6^ per well in 6-well plates. The cells were infected with BCG at an MOI of 10 for 12 h [22]. MOI 10 and 12 h were selected according to our previously published protocol [22], in which these conditions were validated for this macrophage–BCG infection model.

### 2.3. Molecular Docking Validation

The 2D structure of resveratrol (PubChem CID: 445154) was downloaded from the PubChem 2.2 database (https://pubchem.ncbi.nlm.nih.gov 1 October 2026). The 3D structure of the SIRT1 protein (PDB ID: 4I5I; resolution: 2.50 Å) was retrieved from the RCSB PDB database (https://www.rcsb.org 1 October 2026). Water molecules were removed from the target protein using PyMOL 3.1.0, and hydrogen atoms were added to the receptor protein using AutoDock Tools 1.5.6. Resveratrol was set as the docking ligand. During the docking parameter setup, the size of the docking box was adjusted to fully cover the entire protein domain. Docking calculations were performed using AutoDock Vina 1.2.5. The optimal binding conformation was selected based on binding energy and hydrogen bonds (hydrogen bond distance ≤ 3.5 Å). Finally, analysis and visualization were carried out using PyMOL 3.1.0.

### 2.4. Drug Treatment

Resveratrol (Merck, Darmstadt, Germany) was dissolved in DMSO to prepare a 20 mM stock solution, aliquoted into 20 µL/tube, and diluted to 10 μM (low), 20 μM (medium), and 40 μM (high) concentrations before use. EX527 (Purchased from AmBeed, Arlington Heights, IL, USA) was dissolved in DMSO to prepare a 10 mM stock solution, aliquoted into 10 µL/tube, and diluted to 10 μM concentrations before use. All aliquoted solutions were stored at −80 °C. The final DMSO concentration in all cell culture experiments was 0.1% (*v*/*v*). An equivalent volume of DMSO (0.1%, *v*/*v*) was added to the vehicle control group. The same DMSO concentration (0.1%) was maintained across all treatment groups to exclude any solvent-related effects.

### 2.5. qRT-PCR Detection of SIRT1 mRNA Expression

Total RNA was extracted from the cells using the TRIzol method (TRIzol reagent was purchased from TaKaRa, Shiga, Japan). Using the extracted total RNA as a template and oligo (dT) as a primer, cDNA was synthesized by reverse transcription. β-actin was used as the internal reference gene for detection. A 20 μL reaction system was used: 10 μL SYBR^®^ Premix Ex Taq (2×) (purchased from TaKaRa), 0.8 μL upstream primer (sequences: 5′-ATGACGCTGTGGCAGATTGTT-3′), 0.8 μL downstream primer (sequences: 5′-CCGCAAGGCGAGCATAGAT-3′), 1 μL cDNA, and 7.4 μL ddH_2_O 7.4. The primers for qRT-PCR validation were synthesized by Sangon Biotech (Shanghai, China).

### 2.6. Western Blot

Total cellular protein was extracted using a Whole Protein Extraction Kit (Nanjing KeyGen Biotech Co., Ltd., Nanjing, China) and quantified via a BCA kit (Thermo Fisher, Waltham, MA, USA). Equal amounts of protein samples were separated by SDS-PAGE and subsequently transferred to a PVDF membrane using the wet transfer method. The membrane was blocked with 5% skim milk (Beyotime Biotechnology, Shanghai, China) at room temperature for 2 h, followed by incubation with specific primary antibodies at 4 °C overnight and then with an HRP-conjugated secondary antibody (Biodragon, Suzhou, China, BF03008) at room temperature for 2 h. Finally, protein bands were visualized using an ECL detection reagent (Guangzhou Hucheng Technology Co., Ltd., Guangzhou, China). The primary antibodies used included: LC3B (A19665), Beclin1 (A21191), Atg5 (A19677), Atg7 (A19604), p62 (A19700), Bcl-2 (A19693), Bax (A19684), Caspase 3 (A19654), Caspase 9 (A18676), PARP1 (A19612) (all from ABclonal, Wuhan, China), Ac-FOXO1 (Thermo, PA5-116749), FOXO1 (Biodragon, RM5634) and β-actin (Abways, Shanghai, China, AB0035).

### 2.7. Immunofluorescence Assay

In the cell climbing slide experiment, 20 mm cell coverslips were placed in a 12-well plate, and 5 × 10^4^ cells were seeded per well. After cell adhesion, the experimental protocol was followed: the medium was discarded and cells were fixed with 4% paraformaldehyde (Wuhan Servicbio Biotechnology Co., Ltd., Wuhan, China) for 20 min, washed three times with 1× PBS, and permeabilized with 0.5% Triton X-100 (Wuhan Servicbio Biotechnology Co., Ltd., China) for 30 min. Subsequently, the cells were incubated with 3% bovine serum albumin (diluted with 1× PBS, Wuhan Servicbio Biotechnology Co., Ltd.) at room temperature for 1 h. The cells were then incubated with primary antibody (diluted 1:200 in 3% BSA) at 37 °C for 2 h, washed three times with 1× PBS, and incubated with Alexa Fluor 488 goat anti-rabbit IgG (Thermo Fisher Scientific, USA, diluted 1:500 in PBS) at 37 °C for 1 h in the dark. The coverslips were mounted with an antifade mounting medium containing DAPI and stored at 4 °C. Observation was performed using a confocal laser scanning microscope (Leica, Buffalo Grove, IL, USA). Proteins were labeled green, and cell nuclei were stained blue with DAPI.

### 2.8. Observation of Autophagosome-like Structures by Transmission Electron Microscopy

Following sample collection, cells were digested with trypsin and collected by centrifugation at 1000 rpm for 5 min. The cell pellets were pre-fixed with 3% glutaraldehyde at 4 °C for 2 h, washed with PBS, and then post-fixed with 1% osmium tetroxide at 4 °C for 2 h followed by another wash. Dehydration was performed using a graded acetone series (30% to 100%, 15 min per concentration, with 100% repeated three times). Infiltration was carried out sequentially with mixtures of dehydrating agent and Epon812 resin at ratios of 3:1, 1:1, and 1:3 (15 min each), followed by embedding in pure Epon812. Ultrathin sections (60–90 nm) were prepared and collected on copper grids. The sections were stained with uranyl acetate at room temperature for 10–15 min and counterstained with lead citrate for 1–2 min. Images were acquired using a JEM-1400FLASH transmission electron microscope (JEOL, Tokyo, Japan).

For quantification of autophagic vesicles, 15 randomly selected cells per experimental group from three independent biological replicates were analyzed. Autophagosomes were identified as double-membrane vesicles containing intact or partially degraded cytoplasmic material, whereas autolysosomes were identified as single-membrane vesicles containing degraded or electron-dense material. The numbers of autophagosomes and autolysosomes per cell were counted, and the total number of autophagic vesicles (autophagosomes + autolysosomes) per cell was calculated.

### 2.9. Detection of Intracellular Autophagy Rate

The CYTO-ID^®^ Autophagy Detection Kit (Enzo Life Sciences, Lausen, Switzerland) was used. This dye specifically stains pre-autophagosomes, autophagosomes, and autophagolysosomes, exhibiting bright green fluorescence without staining lysosomes. Cells were collected, digested with trypsin, and centrifuged at 1000 rpm for 5 min, followed by two washes with PBS. The CYTO-ID^®^ dye was diluted in 1× Assay Buffer at a ratio of 1 µL/mL, and 250 µL of this solution was used to resuspend the cell pellet. The cells were incubated at 37 °C in the dark for 30 min with occasional mixing. After centrifugation, the cells were washed twice with 1× Assay Buffer, resuspended, and analyzed using the FL1 channel of a flow cytometer.

Analytical method: cells were first gated on FSC-A vs. SSC-A to exclude debris, followed by FSC-A vs. FSC-H gating to exclude doublets, and finally gated on single live cells. CYTO-ID^®^ fluorescence was acquired in the FITC channel. The autophagy rate was calculated as the percentage of CYTO-ID^®^-positive cells relative to the total gated cell population, with the positive threshold determined using an unstained control.

### 2.10. Detection of Apoptosis Rate by Annexin V-FITC/PI Dual Staining Flow Cytometry

The apoptosis rate was detected using an Annexin V-FITC/PI Apoptosis Detection Kit (Nanjing KeyGen Biotech Co., Ltd., China) according to the manufacturer’s instructions. In addition to the experimental groups, three control groups were set up: blank control, Annexin V single stain, and PI single stain. After being washed with PBS, digested with trypsin, and neutralized with complete medium, the cells were centrifuged, and the supernatant was discarded. The cell pellet was resuspended in 500 μL of Binding Buffer. The blank control group received no dye. The single-stain groups were supplemented with 5 μL of Annexin V-FITC or 5 μL of PI, respectively. The experimental group was sequentially stained with 5 μL of Annexin V-FITC and 5 μL of PI. All samples were incubated at room temperature in the dark for 20 min. After centrifugation and removal of the supernatant, the cells were resuspended in 500 μL of Binding Buffer and analyzed by flow cytometry. Apoptosis rate was depicted in a quadrant plot (Q1 and Q4 quadrants are necrotic and viable cells, and Q2 and Q3 quadrants are late apoptotic and early apoptotic cells, Q2 + Q3 = apoptosis rate). All flow cytometry experiments were performed with three independent biological replicates (*n* = 3). The representative plots shown in the figures are from one representative experiment, whereas the bar graphs present the mean ± SD of all three biological replicates. Statistical significance was determined by one-way analysis of variance (one-way ANOVA).

### 2.11. Determination of Resveratrol Toxicity on Macrophage RAW264.7 Cells

Cells were seeded into a 96-well plate at a density of 5000 cells per well (100 μL) and pre-cultured for 24 h at 37 °C in a 5% CO_2_ incubator. After adherence, different concentrations of resveratrol (0.0 μM, 2.5 μM, 5.0 μM, 10.0 μM, 20.0 μM, 40.0 μM, 80.0 μM, 100.0 μM, 150.0 μM, and 200.0 μM) were added to the respective wells. After an additional 6 h of incubation, 10 μL of CCK-8 solution was added to each well, followed by a 2 h incubation period. The absorbance was then measured at 450 nm. Cell viability (%) was calculated as follows: [(OD of experimental group − OD of blank)/(OD of control group − OD of blank)] × 100%.

### 2.12. Statistical Analysis

All data in this study were verified by three independent experiments. The grayscale values of Western blot bands were obtained using Image J software (version 2019) and normalized relative to or β-actin. Experimental data were statistically analyzed by one-way ANOVA using GraphPad Prism 9.0 software (GraphPad Software, San Diego, CA, USA). Data were analyzed using one-way ANOVA followed by Tukey’s post hoc test for multiple comparisons to determine statistical significance. Results are expressed as mean ± standard deviation (X ± SD). Significance was denoted as ns: *p* > 0.05, *: *p* < 0.05, **: *p* < 0.01, and ***: *p* < 0.001.

Regarding the choice of β-actin rather than GAPDH, β-actin was selected because it is a structural cytoskeletal protein whose expression is not directly coupled to cellular metabolic pathways. In contrast, GAPDH is a key glycolytic enzyme, and its expression can fluctuate under conditions that alter cellular metabolism or stress status, which are precisely the conditions involved in our experimental model (RES treatment and autophagy/apoptosis analysis). Therefore, β-actin provides a more stable and reliable reference under our experimental conditions.

## 3. Results

### 3.1. Molecular Docking Validation of RES with SIRT1

**Figure 1:**
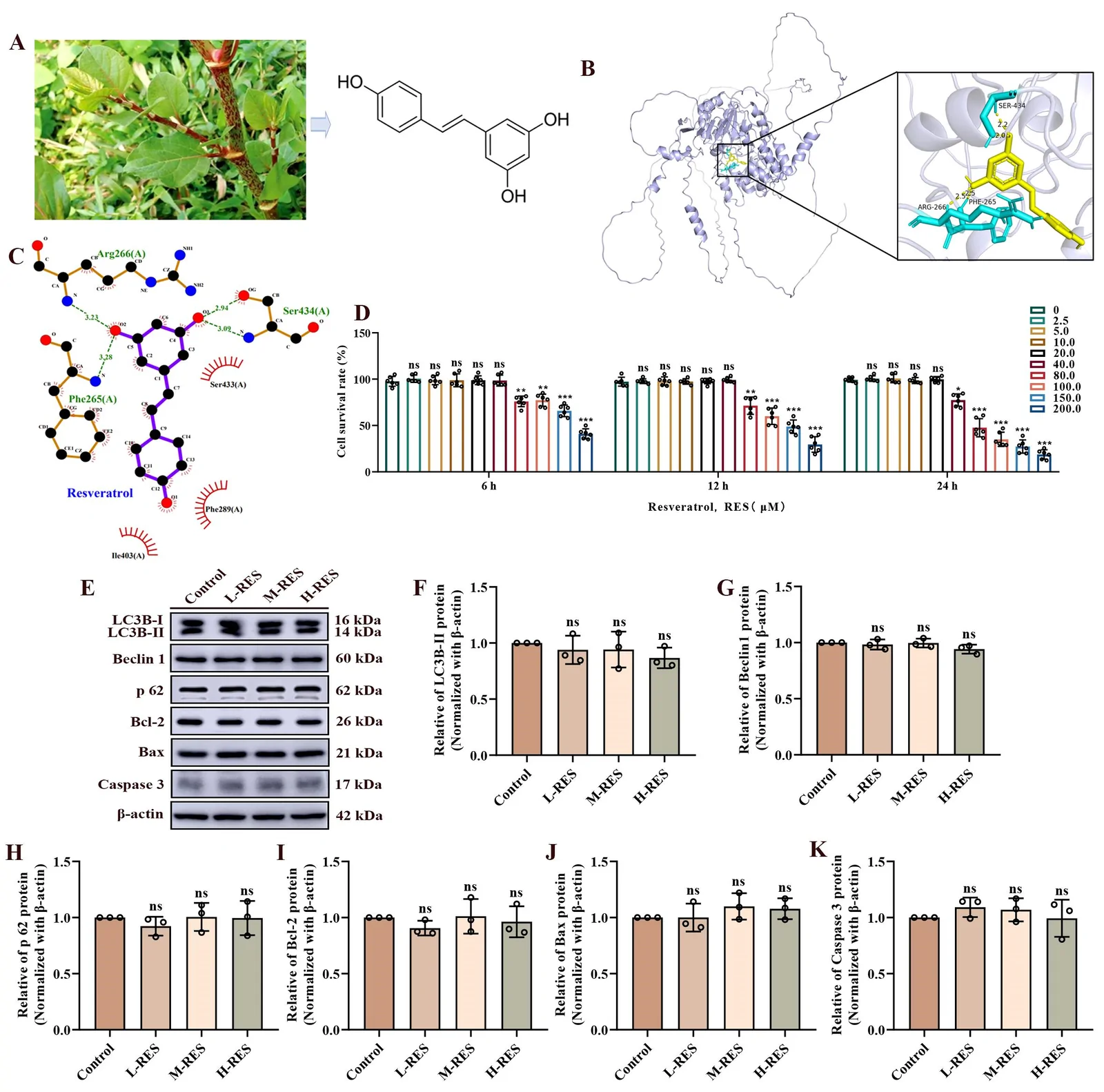
Effects of RES on RAW264.7 cell viability and validation of its own effects (*n* ≥ 3). (**A**). Natural source of resveratrol—*Polygonum cuspidatum*—and the chemical structural formula of RES. (**B**). Three-dimensional conformational diagram of molecular docking between RES and the target molecule SIRT1 (binding affinity: −7.32 kcal/mol). (**C**). Two-dimensional conformational diagram of molecular docking between RES and the target molecule SIRT1 (binding affinity: −7.32 kcal/mol). (**D**). Effect of RES on the viability of RAW264.7 cells. (**E**). Representative Western blot images of key autophagy and apoptosis proteins in RAW264.7 cells. (**F**–**K**). Relative protein expression levels of LC3B-II, Beclin1, p62, Bcl-2, Bax, and Caspase 3. Data were analyzed using one-way ANOVA followed by Tukey’s post hoc test for multiple comparisons to determine statistical significance. Results are expressed as mean ± standard deviation (X ± SD). Significance was denoted as ns: *p* > 0.05, *: *p* < 0.05, **: *p* < 0.01, and ***: *p* < 0.001.

Polygonum cuspidatum is rich in RES and serves as the primary raw material for the industrial extraction of RES (Figure 1A). To investigate the specific molecular interactions between RES and SIRT1, we performed an analysis using molecular docking simulations. The docking model revealed (Figure 1B,C) that hydrogen bond interactions were formed between SIRT1 and RES at the amino acid residues SER-434, ARG-266, and PHE-265, with a binding energy of −7.32 kcal/mol. The molecular docking results indicate a strong binding relationship between RES and the SIRT1 target, suggesting that SIRT1 is a potential target of RES.

It should be noted that the molecular docking analysis presented here was intended as a preliminary exploration of potential binding modes between RES and SIRT1, rather than as experimental proof of direct binding. The predicted binding energy and interacting residues suggest a plausible binding pose; however, these results are computational predictions only. Definitive evidence of direct RES-SIRT1 binding would require experimental validation such as SPR, ITC, or CETSA, which is acknowledged as a limitation and will be pursued in future studies.

### 3.2. Effect of RES on the Viability of RAW264.7 Cells and Validation of Its Basal Effects

The impact of RES on RAW264.7 cell viability was detected using the CCK-8 assay. The results (Figure 1D) showed that cell viability decreased significantly after 6 h of treatment at concentrations greater than 40.0 μM (*p* < 0.01), after 12 h of treatment at concentrations greater than 40.0 μM (*p* < 0.01), and after 24 h of treatment at concentrations greater than 20.0 μM (*p* < 0.05). Based on this, concentrations of 10.0 μM, 20.0 μM, and 40.0 μM with 6 h of treatment were selected for subsequent experiments as low (L-RES), medium (M-RES), and high (H-RES) doses, respectively. To rule out any intrinsic activating effect of RES on macrophages, Western blot analysis was performed. No significant differences (*p* > 0.05) were observed in the expression levels of autophagy-related proteins (LC3B-II, Beclin1, p62) or apoptosis-related proteins (Bcl-2, Bax, Caspase 3) between any dose group and the control group (Figure 1E–K). This indicates that RES alone did not significantly alter basal autophagy/apoptosis under our experimental conditions (*n* = 3), providing a reliable foundation for subsequent studies on its protective effects.

### 3.3. RES Enhances BCG-Induced Autophagosome Accumulation in RAW264.7 Cells

**Figure 2:**
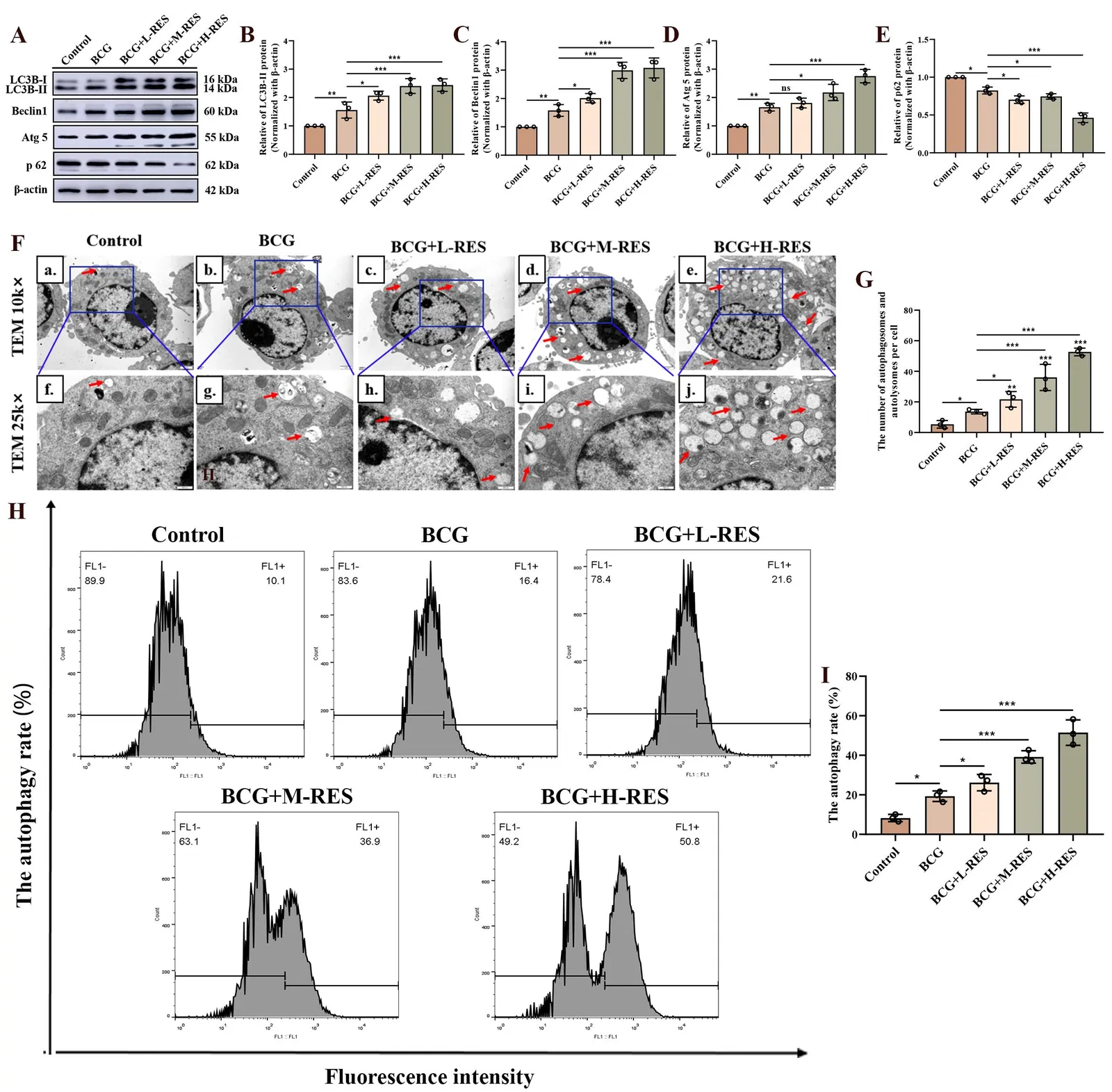
RES enhances BCG-induced autophagy levels in RAW264.7 cells (*n* ≥ 3). (**A**). Representative Western blot images of key autophagy-related proteins in RAW264.7 macrophages. (**B**–**E**). Relative protein expression levels of LC3B-II, Beclin1, Atg5, and p62. (**F**). Observation of autophagosomes and autolysosomes in RAW264.7 cells by transmission electron microscopy (10k×, 25k×; red arrows indicate autophagosomes and autolysosomes, where autophagosomes are double-membrane vesicles containing intact intracellular material, while autolysosomes are single-membrane vesicles containing degraded high-electron-density contents, for quantification of autophagic vesicles, 15 randomly selected cells per experimental group from three independent biological replicates were analyzed). (**G**). Statistical histogram of autophagosomes and autolysosomes. (**H**). Autophagy rate in RAW264.7 cells detected by CYTO-ID^®^ staining. (**I**). Statistical histogram of autophagy rate. Data were analyzed using one-way ANOVA followed by Tukey’s post hoc test for multiple comparisons to determine statistical significance. Results are expressed as mean ± standard deviation (X ± SD). Significance was denoted as ns: *p* > 0.05, *: *p* < 0.05, **: *p* < 0.01, and ***: *p* < 0.001.

To investigate the effect of RES on autophagosome levels in BCG-infected macrophages, this study employed multiple experimental techniques for a comprehensive analysis. Western blot results (Figure 2A–E) showed that compared to the Control group, BCG infection significantly upregulated the expression of LC3B-II, Beclin1, and Atg5 (*p* < 0.01) and decreased p62 levels (*p* < 0.05). Compared with the BCG group, treatment with different doses of RES (L-RES, M-RES, H-RES) combined with BCG further upregulated the expression of LC3B-II and Beclin1 (L-RES group *p* < 0.05, M-RES group and H-RES groups *p* < 0.001) and inhibited p62 expression (L-RES group and M-RES group *p* < 0.05, H-RES groups *p* < 0.001). Notably, L-RES had no significant effect on Atg5 expression, whereas M-RES and H-RES also significantly increased Atg5 levels (M-RES group *p* < 0.05, H-RES groups *p* < 0.001).

Observation by transmission electron microscopy (Figure 2F,G) revealed a significant increase in the number of autophagosomes and autolysosomes within cells after BCG infection (*p* < 0.05), and this number was further elevated in all RES treatment groups (L-RES group *p* < 0.05; M-RES and H-RES groups *p* < 0.001). Consistent results were obtained from flow cytometric analysis of the autophagy rate (Figure 2H,I), which indicated that BCG infection increased the autophagy rate (*p* < 0.05), and RES treatment further significantly enhanced this effect (L-RES group *p* < 0.05; M-RES and H-RES groups *p* < 0.001). In summary, RES promotes BCG-induced autophagosome accumulation in macrophages in a dose-dependent manner.

### 3.4. RES Attenuates BCG-Induced Apoptosis Levels in RAW264.7 Cells

**Figure 3:**
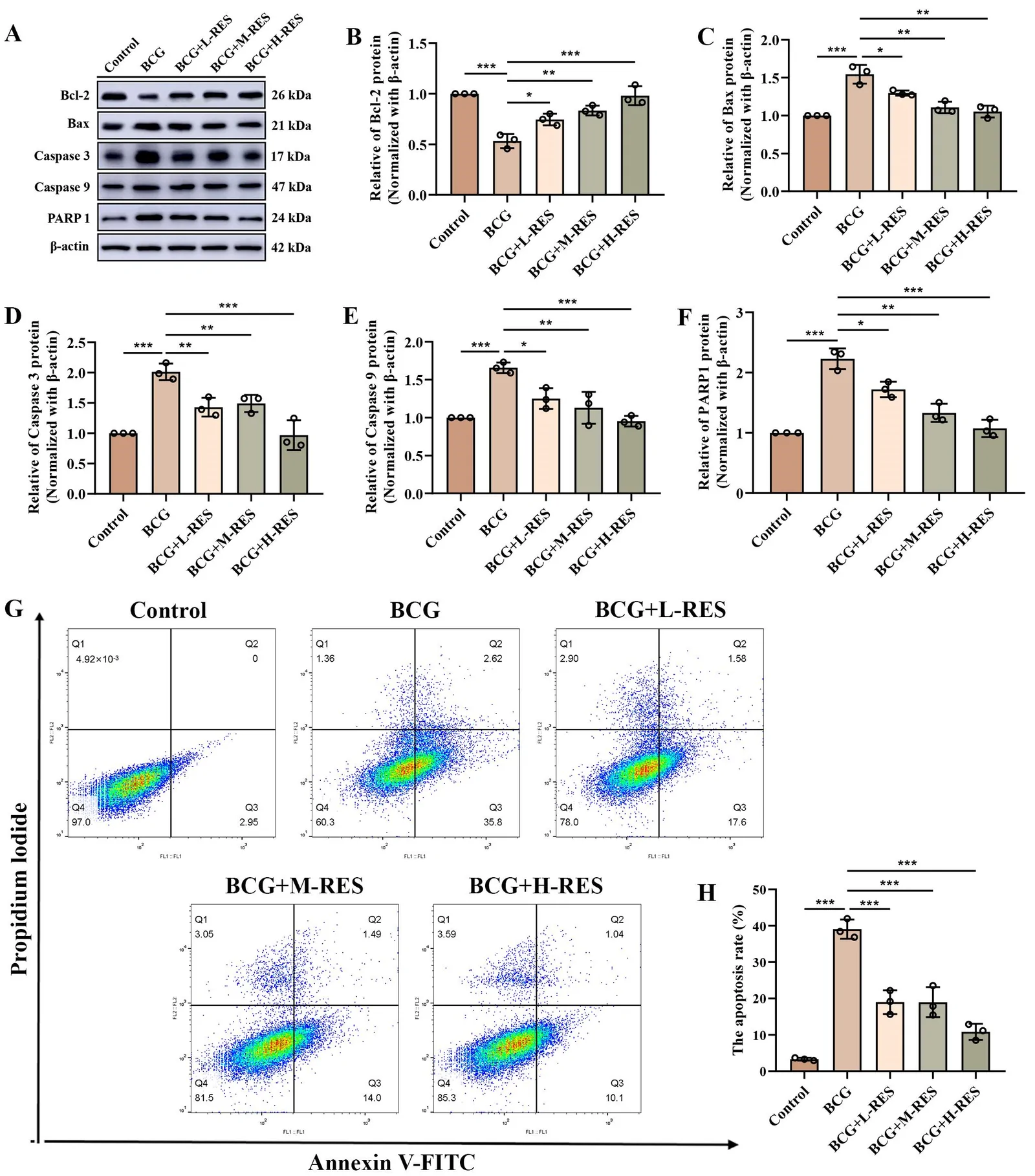
RES reduces BCG-induced apoptosis in RAW264.7 cells (*n* ≥ 3). (**A**). Representative Western blot images of key apoptosis-related proteins in RAW264.7 macrophages. (**B**–**F**). Relative protein expression levels of Bcl-2, Bax, Caspase 3, Caspase 9, and PARP1. (**G**). Macrophage RAW264.7 Apoptosis rate quadrant plot (Q1 and Q4 quadrants are necrotic and viable cells, and Q2 and Q3 quadrants are late apoptotic and early apoptotic cells, Q2 + Q3 = apoptosis rate). (**H**). Histogram of macrophage RAW264.7 apoptosis rate. Representative flow cytometry plots are from one representative experiment, whereas bar graphs show the mean ± SD of three independent biological replicates. Statistical significance was determined using one-way ANOVA followed by Tukey’s post hoc test for multiple comparisons. Data are expressed as mean ± standard deviation (mean ± SD). Significance is denoted as follows: *, *p* < 0.05; **, *p* < 0.01; ***, *p* < 0.001.

To investigate the effect of RES on apoptosis levels in BCG-infected macrophages, this study employed multiple experimental techniques for a comprehensive analysis. Western blot results (Figure 3A–F) showed that compared to the Control group, BCG infection significantly upregulated the expression of Bax, Caspase 3, Caspase 9, and PARP1 (*p* < 0.001) and decreased Bcl-2 levels (*p* < 0.001). Compared to the BCG group, co-treatment with different doses of RES (L-RES, M-RES, H-RES) and BCG attenuated the expression of Bax, Caspase 3, Caspase 9, and PARP1 (L-RES group *p* < 0.05 and *p* < 0.001, M-RES group *p* < 0.01 and H-RES groups *p* < 0.01 and *p* < 0.001) and enhanced Bcl-2 expression (L-RES group *p* < 0.05, M-RES group *p* < 0.01 and H-RES groups *p* < 0.001).

Consistent results were obtained from flow cytometric detection of the apoptosis rate (Figure 3G,H): BCG infection significantly increased the cell apoptosis rate (Annexin V+: early apoptosis 35.8% and late apoptosis 2.62% vs. early apoptosis 2.95% and late apoptosis zero, *p* < 0.001), while RES treatment significantly reversed this BCG-induced pro-apoptotic effect. The apoptosis rate decreased to 19.18% (early apoptosis 17.6% and late apoptosis 1.58%) in the L-RES+BCG group (vs. 38.42% in the BCG group, *p* < 0.001), further dropped to 15.49% (early apoptosis 14% and late apoptosis 1.49%) in the M-RES+BCG group (vs. 38.42% in the BCG group, *p* < 0.001), and reached 11.14% (early apoptosis 10.1% and late apoptosis 1.04%) in the H-RES+BCG group (vs. 38.42% in the BCG group, *p* < 0.001). In summary, RES inhibits BCG-induced macrophage apoptosis in a dose-dependent manner.

### 3.5. Effect of Different BCG Infection Durations on the Intracellular Bacterial Load in RAW264.7 Cells

**Figure 4:**
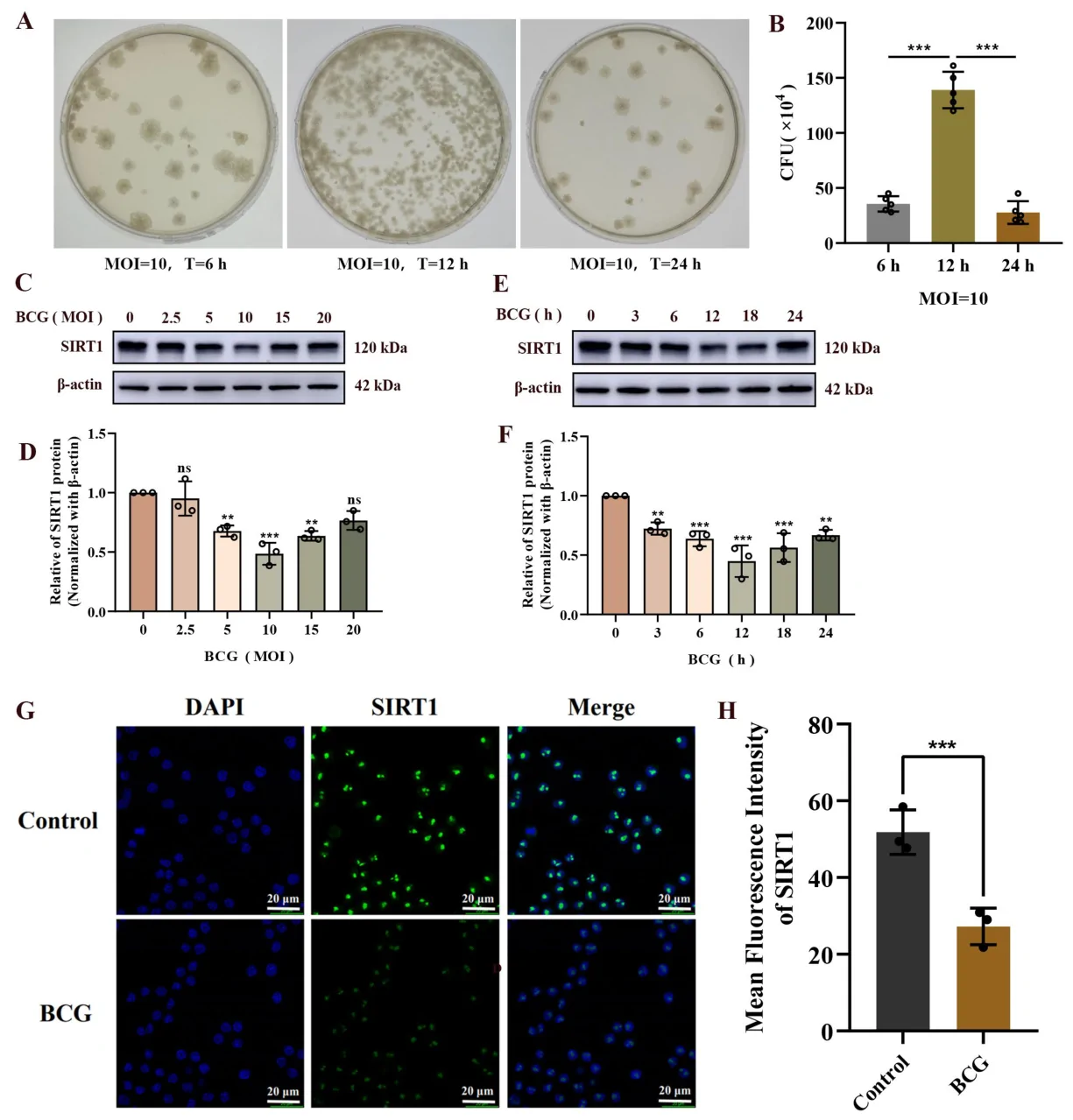
Bacterial load and SIRT1 protein expression in BCG-infected RAW264.7 cells (*n* ≥ 3). (**A**,**B**). Schematic diagram of bacterial load plating and statistical chart of colony counts in RAW264.7 cells after BCG infection (multiplicity of infection MOI = 10, time T = 6 h, 12 h, and 24 h). (**C**). Representative Western blot images of SIRT1 protein in RAW264.7 cells infected with BCG at different concentrations. (**D**). Histogram analysis of SIRT1 protein expression. (**E**). Representative Western blot images of SIRT1 protein in RAW264.7 cells infected with BCG for different durations. (**F**). Histogram analysis of SIRT1 protein expression. (**G**). Immunofluorescence observation of SIRT1 expression in RAW264.7 cells after BCG infection (MOI = 10, T = 12 h). (**H**). Quantitative histogram analysis of immunofluorescence images. Data were analyzed using one-way ANOVA followed by Tukey’s post hoc test for multiple comparisons to determine statistical significance. Results are expressed as mean ± standard deviation (X ± SD). Significance was denoted as ns: *p* > 0.05, **: *p* < 0.01, and ***: *p* < 0.001.

We infected RAW264.7 cells with BCG at a MOI of 10 for 6 h, 12 h, and 24 h, respectively, and screened the optimal infection duration through intracellular bacterial load assays. The results (Figure 4A,B) showed that, as determined by plate colony counting, the bacterial load in cells infected with BCG for 12 h was significantly higher than that at 6 h and 24 h (*p* < 0.001). Therefore, BCG infection of RAW264.7 cells at a multiplicity of infection of 10 for 12 h was selected as the condition for evaluating the drug effects of RES in subsequent experiments.

### 3.6. RES Promotes Upregulation of SIRT1 Expression

To determine the multiplicity of infection (MOI) and infection time that induced the most significant change in SIRT1 protein expression in RAW264.7 cells post-BCG infection, concentration and time gradients were established to identify the optimal conditions. Results showed that SIRT1 expression decreased in a concentration-dependent manner with increasing MOI, reaching its lowest level at an MOI of 10 (*p* < 0.001). Concurrently, SIRT1 protein levels gradually declined over time, with the lowest expression observed at 12 h post-infection (*p* < 0.001) (Figure 4C–F). Based on these findings, subsequent experiments utilized an MOI of 10 and an infection duration of 12 h for BCG infection. Immunofluorescence detection of SIRT1 expression revealed a significant reduction in green fluorescent puncta within cells of the BCG-infected group compared to the control group (*p* < 0.001) (Figure 4G,H). In summary, to ensure cells were under appropriate infectious pressure while achieving the most pronounced change in SIRT1 expression, the optimal conditions for BCG infection of RAW264.7 cells were determined as an MOI of 10 and an infection time of 12 h.

**Figure 5:**
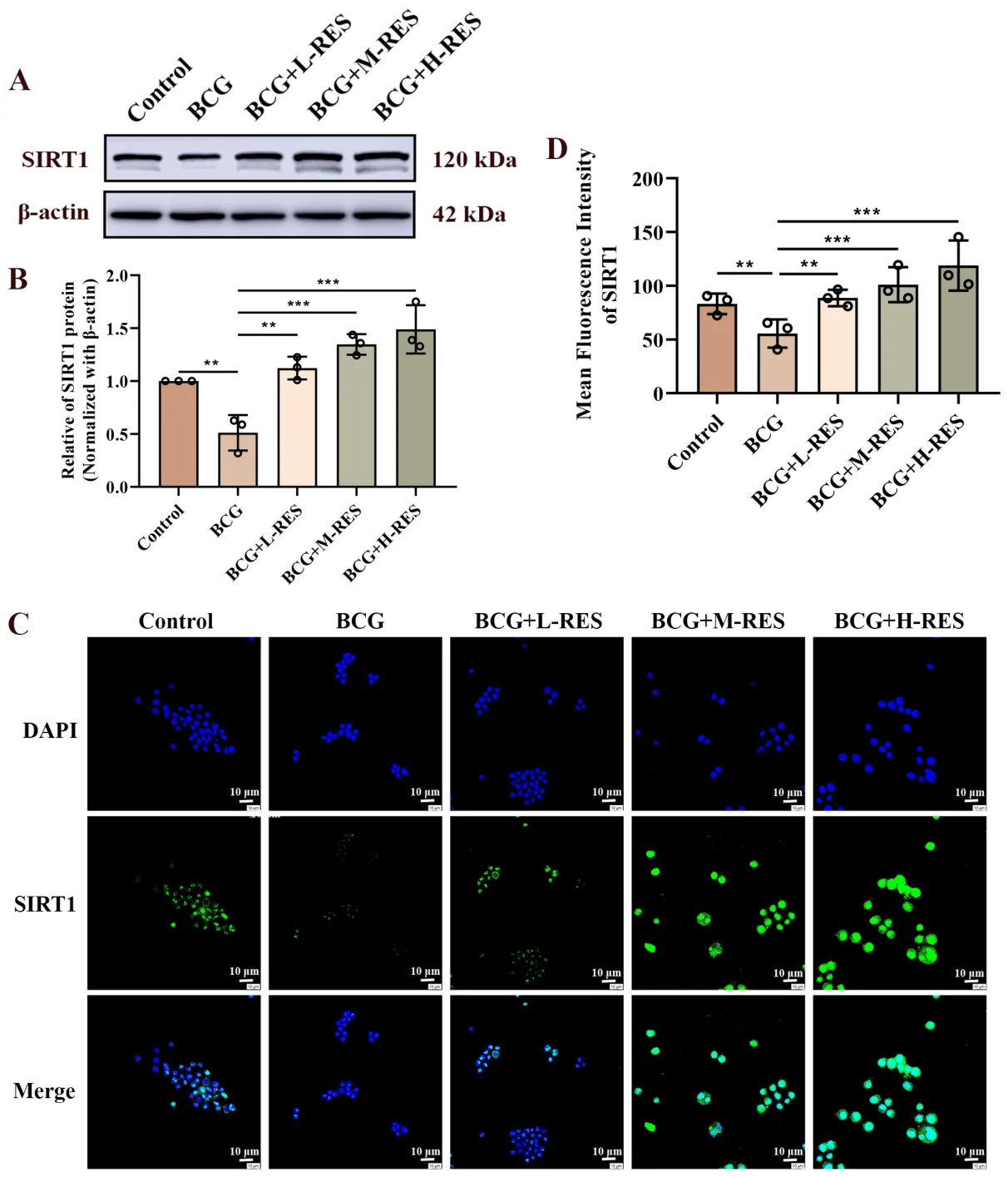
Effect of RES on SIRT1 protein expression in BCG-infected RAW264.7 cells (*n* ≥ 3). (**A**). Representative Western blot images showing the effect of pretreatment with different concentrations of RES on SIRT1 protein in BCG-infected RAW264.7 cells. (**B**). Histogram analysis of SIRT1 protein expression. (**C**). Immunofluorescence observation of the effect of pretreatment with different concentrations of resveratrol on SIRT1 expression in BCG-infected RAW264.7 cells. (**D**). Quantitative histogram analysis of immunofluorescence images. Data were analyzed using one-way ANOVA followed by Tukey’s post hoc test for multiple comparisons to determine statistical significance. Results are expressed as mean ± standard deviation (X ± SD). Significance was denoted as **: *p* < 0.01, and ***: *p* < 0.001.

To investigate the regulatory effect of RES on SIRT1, RAW264.7 cells were pretreated with RES before establishing the BCG infection model. Changes in SIRT1 protein expression were assessed using Western blot and immunofluorescence. Western blot results demonstrated that BCG infection significantly reduced SIRT1 protein levels compared to the Control group (*p* < 0.01). In contrast, cells pretreated with different concentrations of RES prior to BCG infection showed a significant recovery in SIRT1 expression compared to the BCG group (L-RES group *p* < 0.01, M-RES group and H-RES groups *p* < 0.001) (Figure 5A,B). Immunofluorescence results further confirmed that the number of green fluorescent puncta significantly decreased after BCG infection (*p* < 0.01), while RES pretreatment markedly increased the fluorescent signal (L-RES group *p* < 0.01, M-RES group and H-RES groups *p* < 0.001) (Figure 5C,D). These findings, consistent with the protein-level data, indicate that RES effectively reverses BCG-induced suppression of SIRT1 expression and enhances its protein levels.

### 3.7. Design and Validation of SIRT1 Small Interfering RNA

**Figure 6:**
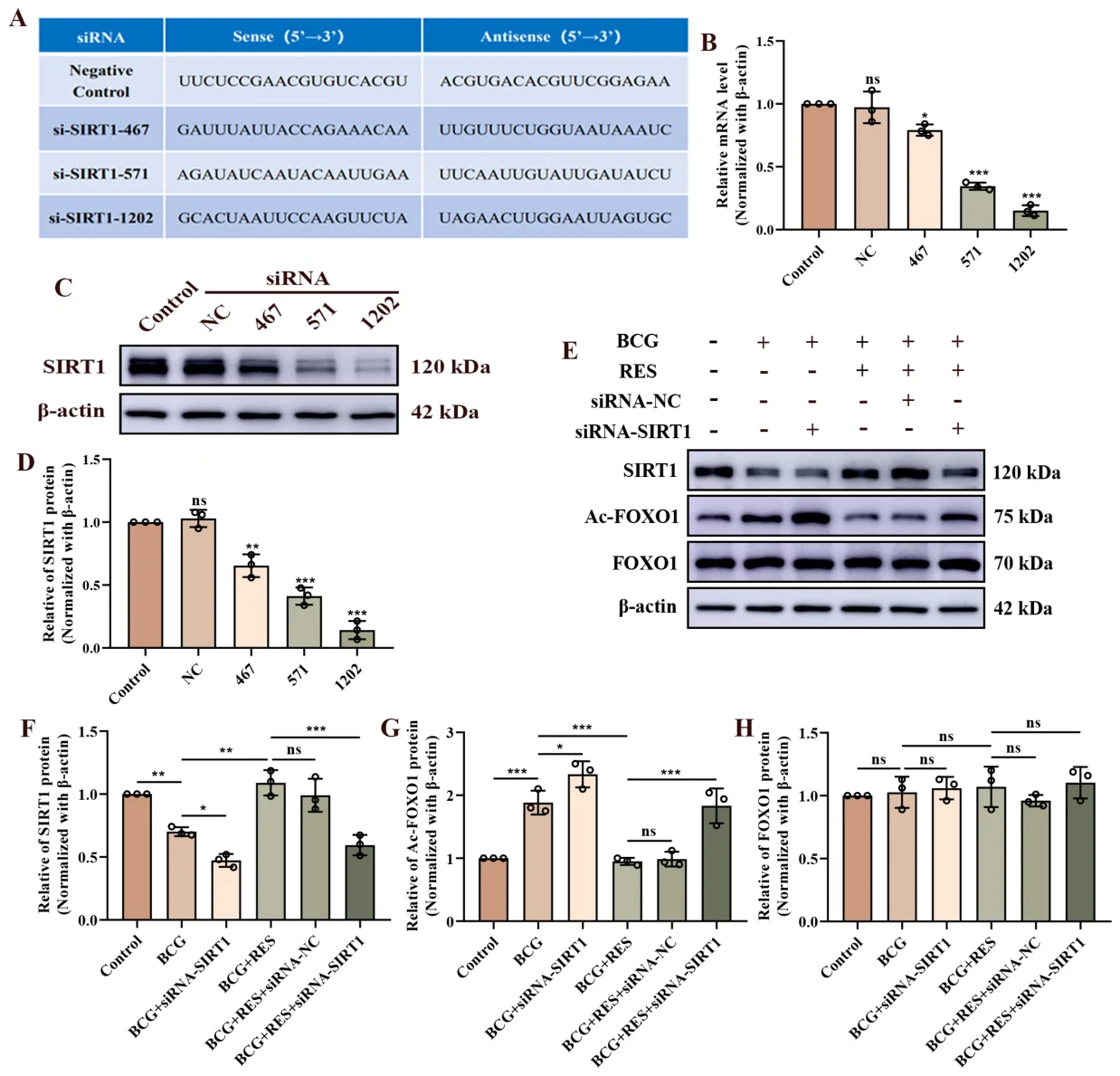
Design and efficacy validation of SIRT1 small interfering RNA (*n* ≥ 3). (**A**). SIRT1 small interfering RNA sequences. (**B**). Histogram of the interference effect of siRNA-SIRT1 at the mRNA level. (**C**). Representative Western blot images of the interference effect of siRNA-SIRT1. (**D**). Histogram analysis of SIRT1 protein expression. (**E**–**H**). Representative Western blot images and corresponding histogram analyses of the effects of siRNA-SIRT1 on the protein expression of SIRT1, Ac-FOXO1, and FOXO1 in RES-pretreated BCG-infected RAW264.7 cells. Data were analyzed using one-way ANOVA followed by Tukey’s post hoc test for multiple comparisons to determine statistical significance. Results are expressed as mean ± standard deviation (X ± SD). Significance was denoted as ns: *p* > 0.05, *: *p* < 0.05, **: *p* < 0.01, and ***: *p* < 0.001.

In previous studies, we found that BCG infection of RAW264.7 cells inhibited SIRT1 protein expression, while RES pretreatment not only alleviated this BCG-induced decrease in SIRT1 expression but also enhanced autophagosome accumulation and suppressed apoptosis. However, whether RES regulated BCG-induced autophagosome accumulation and apoptosis by activating SIRT1 remained unclear. To address this, we further investigated the role of SIRT1 in cellular autophagosome accumulation and apoptosis by inhibiting its expression. Three SIRT1 small interfering RNAs (si-SIRT1) were designed and synthesized, along with a negative control (si-NC) (Figure 6A). After transfection of RAW264.7 cells with siRNA, the interference efficiency of each siRNA was detected at the mRNA and protein levels by qRT-PCR and Western blot, respectively. The results showed that all three siRNAs significantly reduced SIRT1 protein expression in RAW264.7 cells, with siRNA-SIRT1-1202 exhibiting the most pronounced inhibitory effect (*p* < 0.001) (Figure 6B–D), indicating the successful establishment of the SIRT1 knockdown model.

To investigate whether the regulatory mechanism of RES on autophagosome accumulation and apoptosis in BCG-infected RAW264.7 cells is dependent on SIRT1, this study employed si-SIRT1 transfection combined with RES pretreatment and detected SIRT1, Ac-FOXO1 and FOXO1 protein expression levels via Western blot. The results showed that, compared with the control group, BCG infection significantly inhibited SIRT1 protein expression (*p* < 0.01) and markedly promoted Ac-FOXO1 protein expression (*p* < 0.001), while FOXO1 protein expression showed no significant change (*p* > 0.05). Compared with the BCG group, the BCG+si-SIRT1 group showed significantly decreased SIRT1 expression (*p* < 0.05) and significantly increased Ac-FOXO1 expression (*p* < 0.05), while FOXO1 protein expression showed no significant change (*p* > 0.05). In comparison, compared with the BCG group, the BCG+RES group showed significantly increased SIRT1 expression (*p* < 0.01) and significantly decreased Ac-FOXO1 expression (*p* < 0.001), while FOXO1 protein expression showed no significant change (*p* > 0.05). In the RES treatment group, no significant differences in the above three proteins were observed between the BCG+RES+si-NC group and the BCG+RES group (*p* > 0.05). However, in the BCG+RES+si-SIRT1 group, SIRT1 expression was significantly decreased (*p* < 0.001), Ac-FOXO1 expression was significantly increased (*p* < 0.001), while FOXO1 protein expression still showed no significant change (*p* > 0.05) (Figure 6E–H). These results indicate that RES can specifically reverse the decrease in SIRT1 protein expression induced by BCG infection, and further activate the SIRT1/FoxO1 signaling pathway through targeted activation of SIRT1.

### 3.8. RES Promotes BCG-Induced Autophagosome Accumulation in RAW264.7 Cells by Upregulating SIRT1/FOXO1

**Figure 7:**
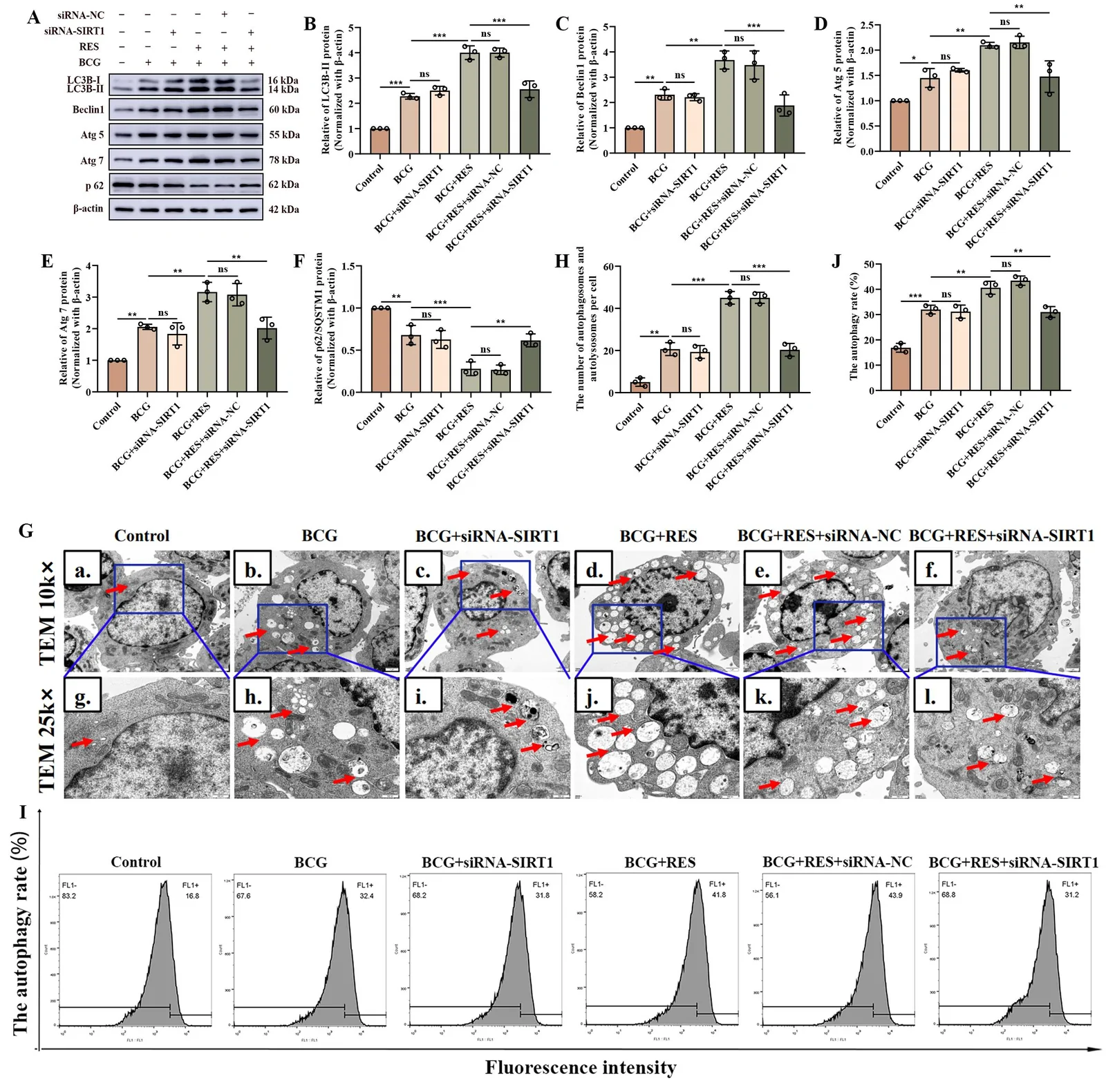
RES promotes autophagy in RAW264.7 cells by upregulating SIRT1/FOXO1 (*n* ≥ 3). (**A**). Representative Western blot images of key autophagy-related proteins in RAW264.7 macrophages. (**B**–**F**). Relative protein expression levels of LC3B-II, Beclin1, Atg5, Atg7, and p62. (**G**). Observation of autophagosomes and autolysosomes in RAW264.7 cells by transmission electron microscopy (10k×, 25k×; red arrows indicate autophagosomes and autolysosomes, where autophagosomes are double-membrane vesicles containing intact intracellular material, while autolysosomes are single-membrane vesicles containing degraded high-electron-density contents, for quantification of autophagic vesicles, 15 randomly selected cells per experimental group from three independent biological replicates were analyzed). (**H**). Statistical histogram of autophagosomes and autolysosomes; (**I**). Autophagy rate in RAW264.7 cells detected by CYTO-ID^®^ staining. (**J**). Statistical histogram of autophagy rate. Data were analyzed using one-way ANOVA followed by Tukey’s post hoc test for multiple comparisons to determine statistical significance. Results are expressed as mean ± standard deviation (X ± SD). Significance was denoted as ns: *p* > 0.05, *: *p* < 0.05, **: *p* < 0.01, and ***: *p* < 0.001.

To investigate whether RES regulates BCG-induced autophagosome accumulation in RAW264.7 cells by activating SIRT1/FOXO1, this study detected the expression of autophagy-related proteins (LC3B-II, Beclin1, Atg5, Atg7, and p62) by Western blot, observed cellular ultrastructure by transmission electron microscopy (TEM), and analyzed the autophagy rate by flow cytometry. The results (Figure 7A–J) showed that compared to the Control group, BCG infection significantly increased the protein expression of LC3B-II (*p* < 0.001), Beclin1 (*p* < 0.01), Atg5 (*p* < 0.05), and Atg7 (*p* < 0.01) while markedly decreasing p62 expression (*p* < 0.01). TEM observation revealed a significant increase in the number of autophagosomes and autolysosomes (*p* < 0.01), and the cellular autophagy rate was also significantly elevated (*p* < 0.001). No significant differences were observed in these indicators between the BCG+si-SIRT1 group and the BCG group (*p* > 0.05). However, compared to the BCG group, the BCG+RES group exhibited further increased expression of LC3B-II (*p* < 0.001), Beclin1 (*p* < 0.01), Atg5 (*p* < 0.01), and Atg7 (*p* < 0.01); a further decrease in p62 expression (*p* < 0.001); and a significant rise in both the number of autophagosomes and autolysosomes (*p* < 0.001) and the autophagy rate (*p* < 0.01). In the RES treatment group, no significant differences in the above indicators were found between the BCG+RES+si-NC group and the BCG+RES group (*p* > 0.05). In contrast, the BCG+RES+si-SIRT1 group showed significantly reduced expression of LC3B-II (*p* < 0.001), Beclin1 (*p* < 0.001), Atg5 (*p* < 0.01), and Atg7 (*p* < 0.01); increased p62 expression (*p* < 0.01); and a marked decrease in both autophagosomes and autolysosomes number (*p* < 0.001) and autophagy rate (*p* < 0.01).

**Figure 8:**
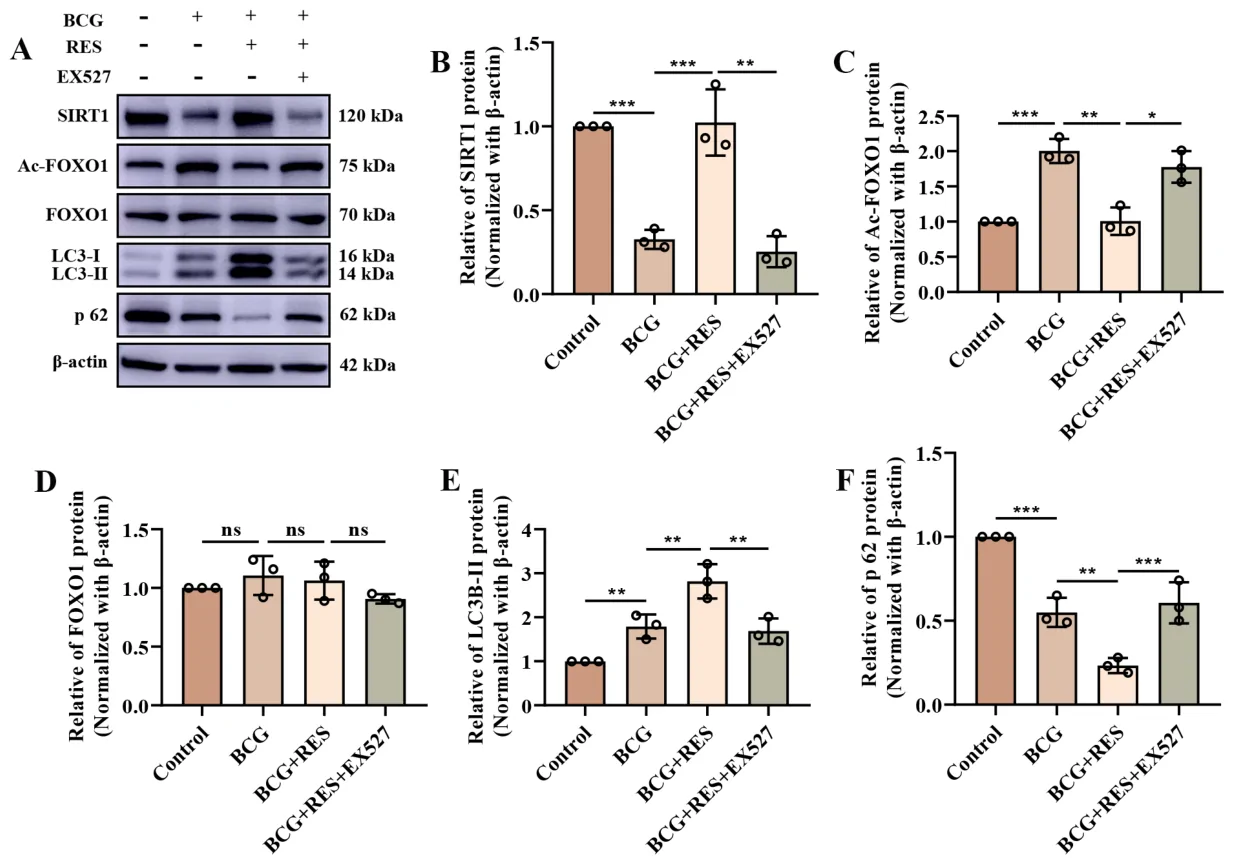
RES promotes autophagy in RAW264.7 cells by upregulating SIRT1/FOXO1 (*n* ≥ 3). (**A**–**F**). Representative Western blot images and corresponding histogram analyses of the effects of the RES inhibitor EX527 on the protein expression of SIRT1, Ac-FOXO1, FOXO1, and autophagy-related proteins LC3B and p62 in RES-pretreated BCG-infected RAW264.7 cells. Data were analyzed using one-way ANOVA followed by Tukey’s post hoc test for multiple comparisons to determine statistical significance. Results are expressed as mean ± standard deviation (X ± SD). Significance was denoted as ns: *p* > 0.05, *: *p* < 0.05, **: *p* < 0.01, and ***: *p* < 0.001.

In addition, we selected the SIRT1 inhibitor EX527 to interfere with the effect of RES on BCG-infected RAW264.7 cells. The SIRT1/FOXO1 pathway and key autophagy proteins were detected by Western blot (Figure 8A–F). The results showed that EX527 intervention in RES treatment significantly reduced the protein expression levels of SIRT1 (*p* < 0.01) and LC3B-II (*p* < 0.01), while the protein expression levels of Ac-FOXO1 (*p* < 0.05) and p62 (*p* < 0.001) were significantly increased and the protein expression level of FOXO1 showed no significant change (*p* > 0.05). These results suggest that RES increases BCG-induced autophagosome accumulation in RAW264.7 cells by activating SIRT1/FOXO1.

### 3.9. RES Inhibits Apoptosis in RAW264.7 Cells by Upregulating SIRT1/FOXO1

**Figure 9:**
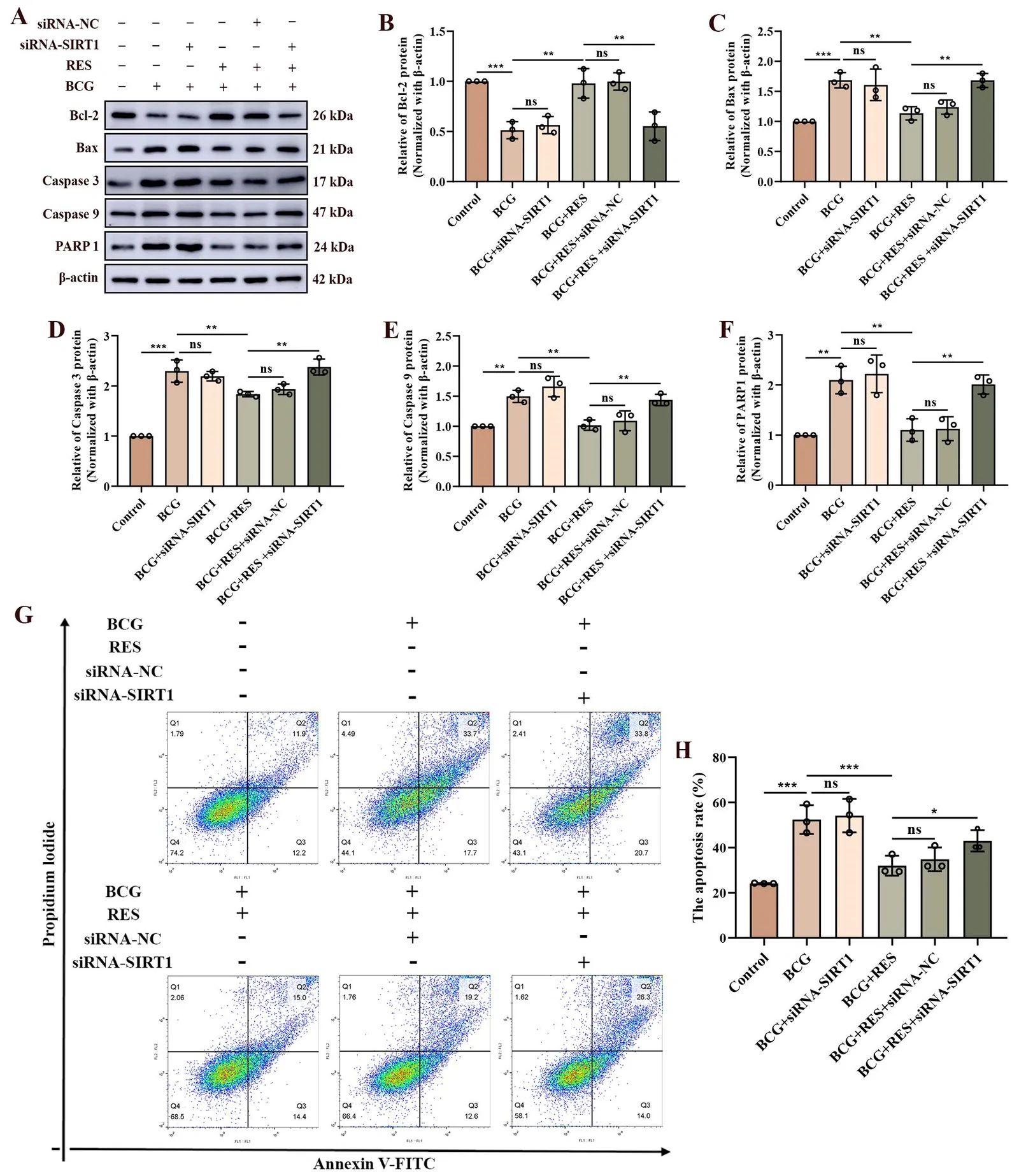
RES inhibits apoptosis in RAW264.7 cells by upregulating SIRT1/FOXO1 (*n* ≥ 3). (**A**). Representative Western blot images of key apoptosis-related proteins in RAW264.7 macrophages. (**B**–**F**). Relative protein expression levels of Bcl-2, Bax, Caspase 3, Caspase 9, and PARP1. (**G**). Macrophage RAW264.7 Apoptosis rate quadrant plot (Q1 and Q4 quadrants are necrotic and viable cells, and Q2 and Q3 quadrants are late apoptotic and early apoptotic cells, Q2 + Q3 = apoptosis rate). (**H**). Histogram of macrophage RAW264.7 apoptosis rate. Representative flow cytometry plots are from one representative experiment, whereas bar graphs show the mean ± SD of three independent biological replicates. Statistical significance was determined using one-way ANOVA followed by Tukey’s post hoc test for multiple comparisons. Data are expressed as mean ± standard deviation (mean ± SD). Significance is denoted as follows: ns, *p* > 0.05; *, *p* < 0.05; **, *p* < 0.01; ***, *p* < 0.001.

To investigate whether RES regulates BCG-induced apoptosis in RAW264.7 cells by activating SIRT1/FOXO1, the apoptotic status was assessed using Western blot to measure the protein expression levels of apoptosis-related factors (Bcl-2, Bax, Caspase 3, Caspase 9, and PARP1) and flow cytometry to determine the apoptosis rate. The results (Figure 9A–H) showed that compared to the Control group, BCG infection significantly inhibited the expression of the anti-apoptotic protein Bcl-2 (*p* < 0.001), while markedly enhancing the expression of the pro-apoptotic proteins Bax (*p* < 0.001), Caspase 3 (*p* < 0.001), Caspase 9 (*p* < 0.01), and PARP1 (*p* < 0.01), and significantly increasing the apoptosis rate (Annexin V+: early apoptosis 17.7% and late apoptosis 33.7% vs. early apoptosis 12.2% and late apoptosis 11.9%, *p* < 0.001). No significant changes were observed in the expression of apoptosis-related proteins or the apoptosis rate (Annexin V+: early apoptosis 20.7% and late apoptosis 33.8% vs. early apoptosis 17.7% and late apoptosis 33.7%) between the BCG+si-SIRT1 group and the BCG group (*p* > 0.05). Compared to the BCG group, the BCG+RES group exhibited significantly increased Bcl-2 expression (*p* < 0.01); decreased expression of Bax, Caspase 3, Caspase 9, and PARP1 (*p* < 0.01); and a markedly reduced apoptosis rate (Annexin V+: early apoptosis 14.4% and late apoptosis 15.0% vs. early apoptosis 17.7% and late apoptosis 33.7%, *p* < 0.001). In the RES-treated groups, no significant differences were found in apoptosis-related protein expression or the apoptosis rate (Annexin V+: early apoptosis 12.6% and late apoptosis 19.2% vs. early apoptosis 14.4% and late apoptosis 15.0%) between the BCG+RES+si-NC group and the BCG+RES group (*p* > 0.05). However, the BCG+RES+si-SIRT1 group showed significantly decreased Bcl-2 expression (*p* < 0.01) and a significant rebound in the expression of Bax, Caspase 3, Caspase 9, and PARP1 (*p* < 0.01), as well as the apoptosis rate (Annexin V+: early apoptosis 14.0% and late apoptosis 26.3% vs. early apoptosis 14.4% and late apoptosis 15.0%, *p* < 0.05). In conclusion, RES inhibits BCG-induced apoptosis of RAW264.7 cells by activating its targets SIRT1/FOXO1.

## 4. Discussion

*Mtb* is a classic intracellular pathogen, and macrophages are its primary target cells [23,24]. After invading the host, *Mtb* is first captured and phagocytosed by alveolar macrophages. *Mtb* evades macrophage eradication by interfering with phagosome formation, allowing it to continue proliferating within the macrophage [25,26,27]. At this stage, *Mtb* encounters a series of dynamic and complex defense systems initiated by the macrophage to inhibit its proliferation and spread, among which autophagy and apoptosis are the two most prominent mechanisms [28,29,30]. Furthermore, *Mtb* can also inhibit macrophage autophagy and apoptosis, leading to programmed necrosis of the macrophage, which provides a favorable opportunity for its own dissemination [31,32]. Thus, the fate of the macrophage not only determines the fate of *Mtb* but also dictates the outcome of tuberculosis.

In recent years, research on autophagy and apoptosis has garnered significant attention. Autophagy, also known as type II programmed cell death, is one of the primary cell death mechanisms within cells. This specific cytological process is crucial for cellular self-digestion, degradation, and maintaining internal homeostasis [33]. It has been reported that autophagy plays a key role in cytoprotection by preventing the accumulation of toxic proteins or modulating the immune system [34]. Apoptosis, or type I programmed cell death, is an active, genetically controlled cell death process during individual development, representing a defensive response to changes in the external environment [35]. The biological mechanisms of apoptosis are consistent with the pathogenic mechanisms of many diseases [36,37]. Recent studies have found that autophagy and apoptosis are closely linked during the development of tuberculosis, and their interaction plays a significant role in the battle between Mtb and macrophages [38]. Some studies report that *Mtb* infection can induce autophagy in host cells [39], and host cell autophagy plays a critical role in countering *Mtb* infection [40]. In this study, after RAW264.7 cells were infected with BCG, numerous autophagosomes appeared intracellularly, and the expression of autophagy-related proteins such as LC3-II, Beclin1, Atg5, and Atg12 was upregulated, while the p62 protein level decreased. These results suggest that BCG promotes autophagosome accumulation in RAW264.7 cells. On the other hand, host cell apoptosis is also closely related to the virulence of *Mtb* [41,42]. Some researchers believe that apoptosis is an important mechanism for macrophages to resist highly virulent *Mtb* strains [43,44]. Apoptotic macrophages can present *Mtb* antigens to CD8+ T cells, thereby enhancing the host’s anti-*Mtb* immune response [45]. Other studies suggest that infection of macrophages with highly virulent *Mtb* strains promotes necrosis, facilitating the spread of infection to other cells, whereas infection with attenuated or mutant strains induces apoptosis, eliminating the invading pathogens [46]. The results of this study show that after BCG infection of RAW264.7 cells, the apoptosis rate increased; expression of Bax, Caspase 3, Caspase 9, and PARP1 rose; and Bcl-2 expression decreased, indicating that BCG promotes apoptosis, which is consistent with the findings mentioned above. However, the specific molecular mechanisms by which BCG infection induces autophagosome accumulation and apoptosis in macrophages remain to be further investigated.

RES is a polyphenolic compound widely found in plants such as grapes, peanuts, and Polygonum cuspidatum. It exhibits significant anti-inflammatory, antioxidant, anti-apoptotic, and immunomodulatory effects [47,48]. Studies have confirmed that RES pretreatment can enhance autophagy activity in human umbilical vein endothelial cells (HUVECs) exposed to cigarette smoke by upregulating the expression of LC3B-II and Beclin1 while suppressing the levels of p62 and mTOR, thereby alleviating apoptosis [49]. Research by Cai et al. [50] found that RES can, at both the transcriptional and translational levels, promote the expression of autophagy-related genes and increase the formation of autophagosomes and autolysosomes, thereby activating autophagy and mitigating H_2_O_2_-induced dysfunction in luteinized granulosa cells in vitro, playing a key role in stabilizing their secretory function and inhibiting apoptosis. Another study by Mohiti et al. [51] demonstrated that kynurenine (KYN) can induce cardiomyocyte apoptosis, while RES prevents KYN-induced apoptosis and concurrently promotes autophagy. Furthermore, it has been reported that resveratrol treatment reduces osteocyte oxidative stress, inhibits osteocyte apoptosis, and promotes autophagy in ovariectomized rats [52]. The results of this study show that pretreatment with low, medium, and high doses of RES enhanced autophagosome accumulation and suppressed apoptosis in BCG-infected RAW264.7 cells. This was manifested by a dose-dependent upregulation of autophagy-related proteins (LC3B-II, Beclin1, Atg5, Atg7) and the anti-apoptotic protein Bcl-2, alongside a downregulation of the autophagy substrate p62 and apoptosis-related proteins (Caspase 3, Caspase 9, Bax, PARP1). These molecular changes ultimately caused a significant increase in the number of autophagosomes and the autophagy rate, while the apoptosis rate decreased. This indicates that RES can promote BCG-induced autophagosome accumulation and inhibit apoptosis in RAW264.7 cells.

SIRT1, an NAD^+^-dependent protein deacetylase, is primarily involved in transcriptional regulation, intracellular energy metabolism, and the regulation of cell death [53,54]. Recent studies have revealed its significant functions in regulating autophagy and apoptosis [55,56]. RES, a recognized specific activator of SIRT1, is thought to exert its effects, at least partially, through SIRT1 activation [57]. One study reported that RES, as a SIRT1 activator, improves the potential for diabetic wound healing by promoting autophagy and inhibiting apoptosis via regulating the SIRT1-FOXO1 axis [58]. It has been reported that RES reduces the vulnerability of the aged heart to ischemia/reperfusion (I/R) injury by inhibiting apoptosis and promoting autophagy through activation of the SIRT1 signaling pathway [59]. Furthermore, research has shown that RES alleviates lead-induced apoptosis in HT-22 cells by activating SIRT1 to inhibit mitochondrial damage and restore lead-impaired autophagic flux [60]. Another study demonstrated that resveratrol exerts a neuroprotective effect on spinal cord injury (SCI) by activating autophagy and inhibiting apoptosis via modulating the SIRT1/AMPK signaling pathway [61]. Our findings are largely consistent with existing reports. The present study found that BCG infection of RAW264.7 cells downregulates SIRT1 protein expression and upregulates Ac-FOXO1 protein expression, accompanied by significant enhancement of autophagosome accumulation and apoptosis. Knocking down SIRT1 expression under these conditions did not significantly affect the levels of autophagosome accumulation or apoptosis, leading to the inference that BCG-induced autophagosome accumulation and apoptosis in RAW264.7 cells may occur independently of the SIRT1/FOXO1 pathway. However, treatment with RES further enhanced autophagic activity and reduced apoptosis, indicating its role in promoting autophagosome accumulation and inhibiting apoptosis. To determine whether the effect of RES depends on the SIRT1/FOXO1 pathway, we knocked down SIRT1 and inhibited SIRT1 with the inhibitor EX527 while administering RES treatment. The results showed that both SIRT1-specific si-SIRT1 and EX527 significantly reversed the effects of RES in promoting autophagosome accumulation and inhibiting apoptosis. This study used the vaccine strain BCG to infect RAW264.7 cells, providing experimental evidence for understanding the pharmacological mechanism of RES. However, whether RES exerts similar effects against virulent *Mtb* strains requires further investigation. Collectively, these findings offer a potential theoretical reference for RES in HDT for tuberculosis. Several limitations of this study should be acknowledged. First, all experiments were performed using the RAW264.7 murine macrophage cell line in vitro. As an immortalized cell line, RAW264.7 cells may not fully recapitulate the phenotypic and functional characteristics of primary macrophages, nor the complex cellular interactions within the in vivo microenvironment. Therefore, although our findings provide mechanistic insights into the regulation of autophagy and apoptosis by RES in BCG-infected macrophages. Future studies using primary macrophages and animal infection models are warranted to validate these findings and to determine their physiological relevance.

However, several limitations of this study should be acknowledged. First, although SIRT1 knockdown was confirmed at both the mRNA and protein levels, a SIRT1 overexpression rescue experiment was not performed. Therefore, while our findings suggest a role for SIRT1 in autophagy and apoptosis regulation under BCG and RES treatment, we cannot fully exclude the possibility of off-target effects of siRNA. Nevertheless, several lines of evidence support the specificity of our observations: the effects of SIRT1 knockdown were phenocopied by the pharmacological SIRT1 inhibitor EX527 and the consistency between siRNA-mediated knockdown and pharmacological inhibition argues against a nonspecific effect. Future studies using SIRT1 overexpression rescue or CRISPR/Cas9-mediated SIRT1 knockout will be important to further confirm the specificity of these findings.

It should be noted that autophagic flux was not directly assessed in the present study; thus, whether the observed autophagosome accumulation reflects enhanced autophagic flux or impaired autophagosome degradation remains to be further investigated. Future studies using tandem fluorescent LC3 reporters are needed to distinguish between these two possibilities. Moreover, whether the increased autophagosome accumulation directly suppresses apoptosis or whether both are independent downstream effects of SIRT1 activation remains unresolved. Future studies using autophagy inhibitors (3-MA or bafilomycin A1) in combination with RES treatment will be needed to dissect this hierarchy.

## 5. Conclusions

This study confirmed that BCG induces autophagosome accumulation and apoptosis in RAW264.7 cells independently of SIRT1. RES promotes BCG-induced autophagosome accumulation and inhibits apoptosis via targeted activation of SIRT1/FOXO1, highlighting the pivotal role of SIRT1 in these effects. However, whether this reflects enhanced autophagic flux or impaired autophagosome degradation requires further investigation.

## Data Availability

The original contributions presented in this study are included in the article material. Further inquiries can be directed to the corresponding authors.
